# Cannabinoids and Cancer

**DOI:** 10.3390/cancers13174458

**Published:** 2021-09-04

**Authors:** Marco Falasca, Mauro Maccarrone

**Affiliations:** 1 Metabolic Signalling Group, Curtin Medical School, Curtin Health Innovation Research Institute, Curtin University, Perth 6102, Australia; 2 Department of Biotechnological and Applied Clinical Sciences, University of L’Aquila, 67100 L’Aquila, Italy; 3 European Center for Brain Research, Santa Lucia Foundation IRCCS, 00164 Rome, Italy

Cannabinoids, active components of the plant *Cannabis sativa,* had been used for centuries in ancient medicine as therapeutic remedies for a variety of conditions, before becoming stigmatized due to their psychoactive effects [1]. In the second half of the 19th century, phyto-cannabinoids have been re-evaluated after the discovery of the chemical structure and isolation of different substances, and the subsequent development of cannabinoid-based drugs that have been FDA approved mainly to treat chemotherapy-induced nausea, insomnia and appetite, epilepsy, spasticity, and pain management [2,3,4]. Then, the elucidation of the endocannabinoid system, from the initial type 1 and type 2 (CB_1_ and CB_2_) cannabinoid receptors and their endogenous ligands (especially *N*-arachidonoylethanolamine, or anandamide, and 2-arachidonoylglycerol) to the emerging complexity of a wider system made up of additional putative receptors, ligands and enzymes, altogether termed *endocannabinoidome*, has further boosted research into the therapeutic potential of phyto-, endo- and even syntho-(synthetic) cannabinoids, cancer treatment included. Unfortunately, despite accumulated evidence pointing in the direction of the potential anti-carcinogenic effects of cannabinoids, there are still few data that corroborate those pre-clinical studies [5], but the fast-paced rhythm of research in this field bodes well for the long-awaited good news [6].

In the current research topic, new important pieces of evidence regarding the role of cannabinoids in different types of cancer and their mechanisms of action are presented in four original manuscripts and six review articles. First, Singh et al. review literature data of cannabinoids’ anti-cancer effects and of their activity as cell proliferation inhibitors, signalling molecules, apoptosis inducers and cell motility deactivators in prostate cancer. The authors concluded that, although several pathways used by cannabinoids to provoke the death of cancer cells have indeed been identified, their mechanism of action remains as yet unclear [7]. Of note, one of those key mechanisms involved in the development and progression of cancer is autophagy. Lee et al. examine the molecular mechanism and role of this complex process in different types of cancer, and the role played by cannabinoids in its regulation. The ambivalent contribution of autophagy to tumour’s spread, inhibitory in early stages through anti-inflammatory and anti-necrosis action and supportive in more advanced stages by supplying energy to cancer cells, is an interesting starting point for innovative therapeutic exploitation. Several studies have demonstrated how cannabinoids, by inducing autophagy, can inhibit cancer cell proliferation in vitro and in some in vivo models through the activation of the p8/TRIB3 pathway; consistently, different cannabinoids in combination with radiotherapy have been found to reduce tumour growth by promoting autophagy; however, these promising data still lack a proper mechanistic understanding and require further investigation [8]. Synthetic cannabinoids WIN55,212-2 and JWH133 were also tested in glioblastoma cells expressing high levels of CB_2_ receptors by Ellert-Miklaszewska et al. in their research article. These authors used five different glioma cell lines and patients-derived cells that were highly resistant to standard chemotherapy due to a lack of tumour suppressor p53 and/or PTEN. Interestingly, the observed cannabinoid-induced autophagy and apoptotic cell death were enhanced by the inactivation of specific autophagy genes, suggesting cannabinoid use as a potential new therapeutic strategy for glioblastoma [9]. The latest tumour is the topic of the work by Kolbe et al., who tested the effects of cannabidiol (CBD) and Δ^9^-tetrahydrocannabinol (THC), the main cannabinoids found in the cannabis plant, on patient-derived glioblastoma cells. Cell cycle analysis revealed a decrease in cell cycle marker Ki67 via G protein-coupled orphan GPR55 receptor upon treatment with THC, pointing to the potential therapeutic action of THC in GPR55-expressing glioblastomas [10]. The review by Andradas et al. takes stock of the situation of cannabinoid’s treatment in paediatric oncology. They report few, and yet only preliminary, results on cannabinoids’ effects on children’s tumours, and unfortunately even less data on their safety and side effects. Remarkably, the few available studies are greatly diversified in terms of methodologies, types of compounds and formulations used, thus leading to controversial observations and a lack of confirmations [11]. Moreover, Andradas et al. reported bad news on this subject in their paper on the role of cannabinoids in the treatment of children’s brain cancers medulloblastoma and ependymoma. Despite positive results in vitro, THC and CBD treatment of mouse models of these tumours failed to have any impact on traditional chemotherapy or on the animals’ survival [12]. Bar-Sela et al. reported unfavourable outcomes for cancer patients, who were cannabis consumers, treated with immunotherapy in their observatory study. Their findings suggest that cannabinoids’ immunomodulatory effects might interfere with immunotherapy and call for caution on their use in those patients [13]. Then, Taylor et al. review the current knowledge of the involvement of endocannabinoid system and its various constituents in reproductive events and in gynaecological cancers. The latter are malignancies originating from either the reproductive tract or the products of conception, or secondary tumours. The review article highlights how targeting the endocannabinoid system could lead to new approaches to the diagnosis and treatment of these cancers in the female reproductive system [14].

Another review analyses the complex involvement of the various components of the endocannabinoid system in susceptibility to cancer, prognosis, and response to treatment, focusing on the relationship of this lipid signalling system with cancer biology in different paradigms, such as gastrointestinal, gynaecological, prostate, thoracic, thyroid, and brain cancers, as well as melanoma. Interestingly, Moreno et al. noted that the same component of the endocannabinoid system can exert both pro- and anti-tumoural actions in different tumour subtypes [15].

Finally, Afrin et al. discuss the action of non-THC cannabinoids in cancer. In their study, these authors evidenced the paucity of clinical data using cannabinoids, and the need for further preclinical investigations in order to establish the ‘pros’ and ‘cons’ of using whole cannabis extracts [16].

Taken together, we hope to offer the reader at least a compass to navigate the mare magnum of the cannabinoid and endocannabinoid literature in the context of cancer.
